# Case Report: Toxic epidermal necrolysis induced by sintilimab in a patient with advanced lung squamous cell carcinoma

**DOI:** 10.3389/fphar.2026.1610305

**Published:** 2026-01-29

**Authors:** Jiaxing Wu, Mingjun Hu, Juan Chen, Qingqun Wang, Ailing Wang, Wanli Mao

**Affiliations:** 1 School of Medicine, Nankai University, Tianjin, China; 2 Department of Pathology, Sino Singapore Cancer Centre, Chongqing, China; 3 Department of Nursing, Sino Singapore Cancer Centre, Chongqing, China; 4 Department of Oncology, Sino Singapore Cancer Centre, Chongqing, China

**Keywords:** case report, immune-related adverse events (irAEs), non-small cell lung cancer, sintilimab, toxic epidermal necrolysis

## Abstract

**Background:**

Sintilimab is an effective PD-1 immune checkpoint inhibitor (ICI) for advanced non-small cell lung cancer (NSCLC). However, it can cause severe immune-related adverse events (irAEs) such as toxic epidermal necrolysis (TEN), a rare hypersensitivity reaction with significant mortality. Reports of Sintilimab-induced TEN are exceedingly rare, making its recognition and management crucial.

**Case summary:**

A 60-year-old female with advanced NSCLC developed TEN 3 days after her second dose of Sintilimab. The condition progressed rapidly, with epidermal detachment affecting 85% of her body surface area (BSA). Immediate interventions, including high-dose corticosteroids, intravenous immunoglobulin, meticulous wound care, and infection control, led to gradual recovery. After 39 days of intensive care, the patient was discharged with complete healing of skin lesions and no significant complications.

**Conclusion:**

This report highlights the potential for Sintilimab to induce life-threatening TEN, emphasizing the need for vigilant monitoring and prompt intervention during ICIs therapy.

## 1 Introduction

Sintilimab, a humanized anti-PD-1 monoclonal antibody, has demonstrated significant efficacy in the treatment of advanced non-small cell lung cancer (NSCLC) by enhancing T-cell immune responses (Zhang et al., 2022). However, similar to other immune checkpoint inhibitors (ICIs), it can cause severe immune-related adverse events (irAEs), including toxic epidermal necrolysis (TEN) (Satoh et al., 2023). TEN is a life-threatening type IV hypersensitivity reaction characterized by widespread epidermal necrosis and detachment, affecting more than 30% of the total body surface area (BSA) (Singh and Phillips, 2022; Hinc-Kasprzyk et al., 2015). TEN can lead to serious complications such as infections, electrolyte imbalances, and organ failure, with a mortality rate of up to 40% (Singh and Phillips, 2022; Hinc-Kasprzyk et al., 2015; Sorrell et al., 2017).

Despite extensive use of Sintilimab in NSCLC management (Zhang et al., 2022), reports of TEN associated with this agent remain exceedingly rare, with only three documented cases in the literature to date (Jiang et al., 2023; Li et al., 2022; Lye et al., 2023) (Supplementary Table S1). This case provides novel insights through: (1) First documentation of TEN after re-initiation of Sintilimab (second exposure a year apart) with the shortest latency period (72 h post-infusion) reported to date; (2) Demonstration of successful management in a patient with up to 85% BSA involvement; (3) Comprehensive longitudinal tracking of mucosal and systemic manifestations. Our experience underscores the imperative for early recognition algorithms and standardized management protocols in ICIs-associated TEN.

## 2 Case presentation

The patient was a 60-year-old Asian female. In February 2023, she was diagnosed with centrally located lung squamous cell carcinomas (T4N2MX). Immunohistochemical staining showed CK (+), BRG1 (+), CK5/6 (+), P40 (+), TTF-1 (−), NapsinA (−), Syn (−), Ki-67 (50% +).

She received four cycles of chemotherapy with paclitaxel and carboplatin, but due to disease progression, the regimen was switched to docetaxel, cisplatin, plus Sintilimab in June 2023. After one cycle of the combined therapy, the patient developed severe gastrointestinal reactions and opted for voluntary discharge. She did not continue with regular follow-up care thereafter. There were no obvious side effects related to rash during the treatment performed above.

On 28 February 2024, the patient was re-admitted with complaints of lower back pain and right lower limb weakness. She was diagnosed with stage IV lung squamous cell carcinoma with liver, bone, and cervical lymph node metastasis (cT4N3M1).

After admission, palliative radiotherapy was administered to the lumbosacral region due to bone metastases, with 13 sessions from March 5 to 21 March 2024. On March 25, palliative radiotherapy was extended to the nasal mass and cervical lymph nodes. The treatment regimen also included oral vinorelbine (60 mg) and Sintilimab (200 mg).

Palliative radiotherapy was administered to the lumbosacral region (March 5–21) and later to the nasal mass and cervical lymph nodes (starting March 25) (Supplementary Table S2). Concurrently, oral vinorelbine (60 mg) and sintilimab (200 mg) were initiated on March 25.

**FIGURE 1:**
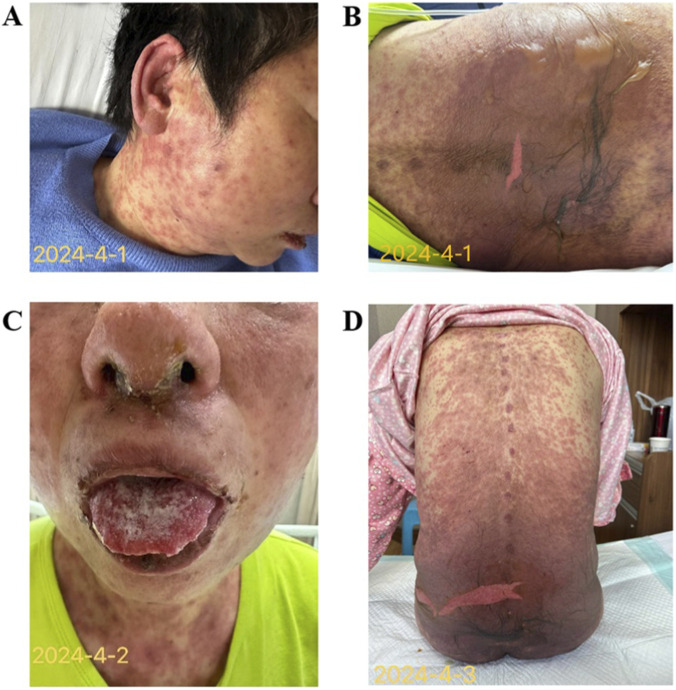
Early-stage rash presentation **(A)** April 1: Scattered erythematous rash over the entire body, with erythema and papules on the face and neck. **(B)** April 1: Confluent rash on the lower back and abdomen with bullae formation; an approximately 6 cm × 2 cm erosion on the lower back. **(C)** April 2: Erosions and crusting on the upper and lower lips and nasal mucosa, with white membrane formation in the oral cavity and on the tongue. **(D)** April 3: Disease progression with increased epidermal detachment.

On March 28, 3 days after receiving Sintilimab, scattered erythematous maculopapular eruptions first appeared on the patient’s lower back and arms. Given the localized distribution and mild nature of the rash, clinical assessment favored continuing the planned palliative radiotherapy. The following day (March 29), the cutaneous eruption progressed, with similar lesions appearing on the face and neck, accompanied by bilateral conjunctival redness, which is a recognized manifestation of ICIs-associated TEN (Ma et al., 2021). At this point, radiotherapy was halted. Subsequently, the patient’s condition deteriorated rapidly, with the rash spreading over most of her body, accompanied by high skin temperature and severe itching (Figures 1A–D).

**FIGURE 2:**
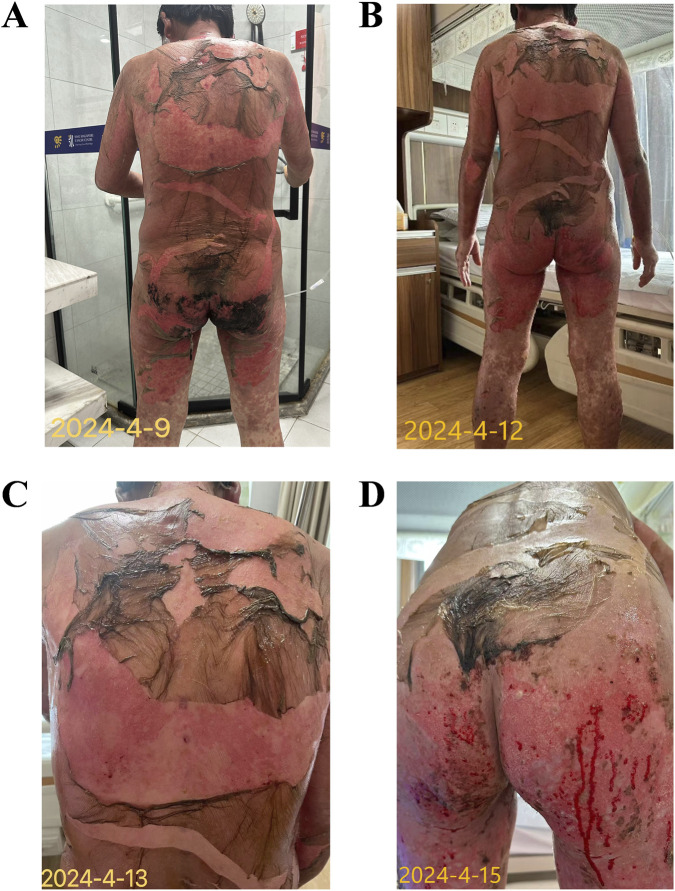
Progressive-stage rash presentation **(A)** April 9: Rash extends to 70%–80% BSA with extensive epidermal detachment (≥18% BSA). **(B)** April 12: Generalized rash, bullae, and epidermal detachment involving approximately 85% BSA; epidermal sloughing accounts for about 35% BSA. **(C)** April 14: Extensive epidermal detachment on the back with red, moist wound bed. **(D)** April 15: Epidermal sloughing on the buttocks and posterior thighs with erythematous, exudative wounds and whitish secretions.

From 4 April 2024, the rash had covered approximately 60% BSA, with large blisters forming and some epidermal detachment affecting about 11% BSA. Methylprednisolone (160 mg/day) and intravenous cefuroxime were administered for anti-inflammatory and infection prevention purposes. The condition continued to deteriorate, with the rash extending to 70%–80% BSA and more extensive epidermal detachment (≥18% BSA) by April 8–9 (Figure 2A), leading to painful oral and nasal mucosal erosion.

From April 10 to April 15, generalized rash, bullae, and epidermal detachment involved approximately 85% BSA, with epidermal sloughing accounting for approximately 35% BSA (Figures 2B–D). Intensive wound care, immunoglobulin therapy, and high-dose corticosteroids were continued. Culture tests from blister fluid and exudates revealed *Escherichia coli* infection, which was treated with meropenem.

**FIGURE 3:**
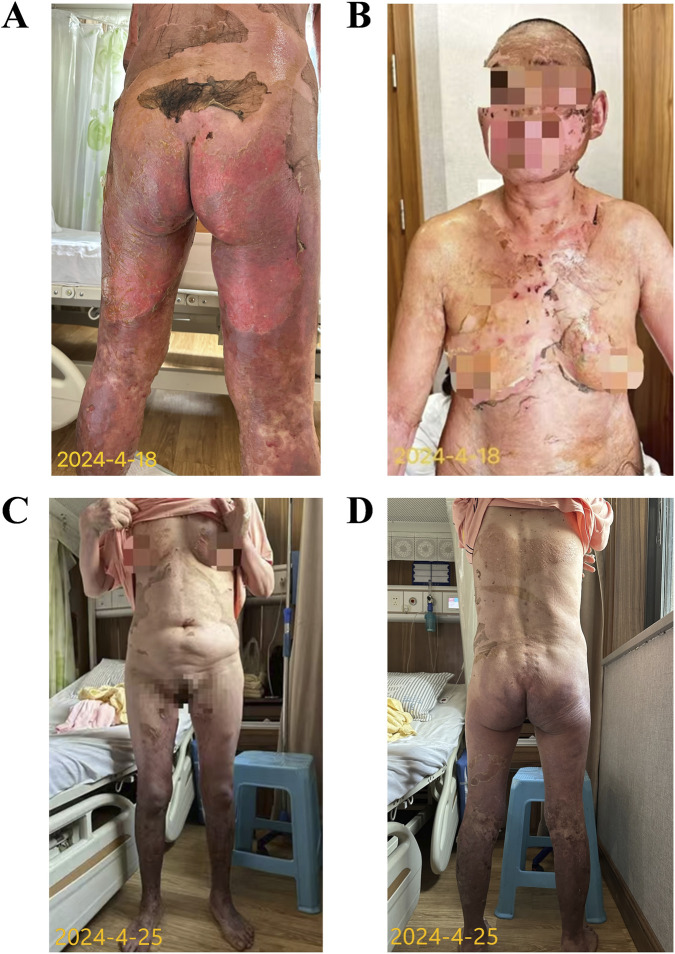
Skin recovering from toxic epidermal necrolysis **(A)** April 18: Wound secretions well controlled, with pink epithelialized skin. **(B)** April 18: Gradual restoration of normal appearance and function of the facial and cervical skin. **(C)** April 25 (Anterior view): Erosions on the trunk and extremities gradually crusted and healed after satisfactory re-epithelialization. **(D)** April 25 (Posterior view): Erosions on the trunk and extremities gradually crusted and healed after satisfactory re-epithelialization.

By April 18 (24 days post-Sintilimab injection), skin redness, edema, and pain gradually improved, and new skin growth was observed on the face, neck, and back (Figures 3A,B). By April 25, the areas of epidermal sloughing had reduced to about 40% BSA, with significant recovery of the original skin appearance and function in many regions (Figures 3C,D). Lower extremity edema persisted but improved with diuretic therapy.

**FIGURE 4:**
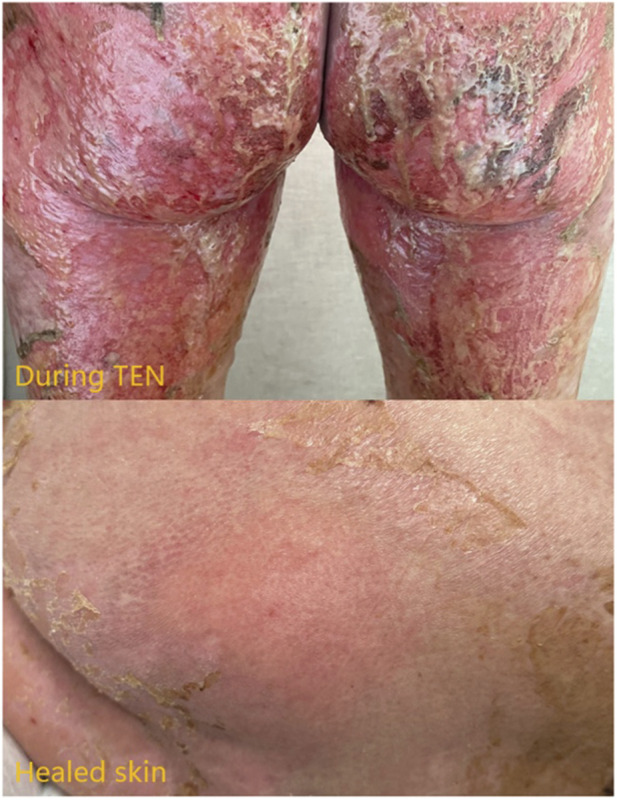
Comparison of buttock skin during TEN and after successful re-epithelialization.

On 6 May 2024 (42 days post-Sintilimab injection), the patient’s skin had almost completely recovered (Figure 4), and she was discharged in stable condition. During a follow-up visit 3 months later, the patient remained in good health with no new skin lesions or long-term complications. A timeline of major treatments during admission is presented in Supplementary Figure S1. Representative CT scans demonstrating tumor changes are presented in Supplementary Figures S2, S3. Changes in major laboratory indicators during admission is presented in Supplementary Table S3.

Informed consent was obtained from the patient before the submission of this case report for publication including the use of images. Ethical approval for this report was obtained from the ethics committee of SINO SINGAPORE CANCER CENTRE.

## 3 Discussion

### 3.1 Clinical presentation and diagnostic evaluation

This case represents a seminal documentation of Sintilimab-induced TEN, a rare immune-related adverse event with mortality rates exceeding 30% in high-risk populations (Sorrell et al., 2017; Zhou et al., 2024). The diagnosis was established through three primary criteria: rapid progression of epidermal detachment affecting over 85% BSA, temporal association with Sintilimab administration (3 days post-second dose), and exclusion of alternative etiologies via comprehensive pharmacovigilance review (Frantz et al., 2021). Although skin biopsy was not performed due to financial constraints, the diagnosis was strongly supported by urgent multi-disciplinary consultations and the characteristic clinical course. Quantitative validation was achieved through the SCORTEN prognostic model (score: 4, predicting a 58.3% mortality risk) (Frantz et al., 2021; Torres-Navarro et al., 2019; Torres-Nav et al., 2020) (Supplementary Table S4) and the ALDEN causality algorithm, which yielded a score of +4 (“probable” association) for Sintilimab (Sassolas et al., 2010) (Supplementary Table S5). A systematic ALDEN-based comparison of all concurrent agents strongly prioritized Sintilimab (high-risk notoriety) over vinorelbine (low-risk) and radiotherapy (atypical cause), effectively strengthening the causal link (Supplementary Table S5). These clinical features met CTCAE 5.0 criteria for grade 4 cutaneous adverse reactions, underscoring the severity of this reaction (Freites-Martinez et al., 2021).

### 3.2 Pathophysiological considerations

The underlying mechanism by which PD-1 inhibitors like Sintilimab can trigger TEN remains incompletely understood but is thought to involve immune dysregulation (Qin et al., 2024). Sintilimab, a recombinant humanized anti-PD-1 monoclonal antibody reported to have high binding affinity (Wang et al., 2019), removes a key inhibitory checkpoint on T-cells by blocking PD-1/PD-L1 signaling (Bhardwaj et al., 2022). In susceptible individuals, this may facilitate the activation of pre-existing, drug-specific cytotoxic CD8^+^ T lymphocytes—the principal effector cells in TEN pathogenesis (Bhardwaj et al., 2022; Shah et al., 2024). These cells, along with neutrophils and other innate immune cells, release a storm of cytotoxic proteins (e.g., granulysin, perforin) and pro-inflammatory cytokines (e.g., TNF-α, IL-6, IL-15), leading to widespread keratinocyte apoptosis (Shah et al., 2024; Saito and Abe, 2023; Zhang et al., 2023). Genetic predisposition, particularly via specific HLA alleles (e.g., *HLA-B*15:02, *HLA-B*58:01), is a well-established risk factor for certain drug-induced SJS/TEN (Shah et al., 2024; Gibson et al., 2023). Although definitive HLA links for ICIs-related TEN are still being defined, such genetic factors likely contribute to individual susceptibility. In this case, the patient developed TEN after re-initiation of Sintilimab (second exposure a year apart) with remarkably rapid onset (72 h), contrasting with prior tolerance during initial therapy. This suggests possible immune priming mechanisms. And the concurrent radiotherapy may have synergistically exacerbated the Sintilimab-triggered immune response through the impairment of regulatory T cells and enhanced immune dysregulation (Satoh et al., 2023; Li et al., 2023).

### 3.3 Management strategies and clinical outcomes

The therapeutic paradigm in this high-mortality case (SCORTEN >4) combined three essential elements besides prompt cessation of suspected causative agents: early immunomodulation with high-dose corticosteroids and intravenous immunoglobulin to counteract cytokine-mediated tissue damage, meticulous barrier protection using protective isolation and targeted wound care, and preemptive antimicrobial management with meropenem for emerging multidrug-resistant infections. This coordinated approach with comprehensive care achieved complete re-epithelialization within 39 days, a timeline surpassing historical outcomes in ICIs-associated TEN.

The patient did not receive further systemic antitumor therapy after discharge. Importantly, a follow-up CT scan revealed significant tumor reduction, indicating a favorable response to Sintilimab (Supplementary Figure S3). Despite this efficacy, all ICIs were permanently discontinued due to the life-threatening TEN, in line with clinical guidelines (Zhou et al., 2021; Ramos-Casals et al., 2020; Haanen et al., 2018). The patient succumbed to malignancy progression on 29 August 2024, with no recurrence of cutaneous symptoms on monthly follow-up until that time. This case underscores the clinical dilemma where profound antitumor activity coexists with prohibitive toxicity.

### 3.4 Implications for immunotherapy practice

Three critical practice implications emerge from this case. First, new-onset mucocutaneous lesions within 72 h of PD-1 inhibitor administration require immediate dermatologic evaluation given the potential for rapid TEN progression. Second, SCORTEN scoring should guide ICU triage decisions, as patients with predicted mortality exceeding 50% benefit from specialized burn unit care. Third, systematic pharmacovigilance frameworks must be implemented to detect rare but lethal adverse events, balancing therapeutic benefits with risk mitigation.

### 3.5 Study limitations

Skin biopsy, HLA genotyping and baseline immune profiling were not performed, primarily due to financial constraints. While such data could have enriched the mechanistic discussion, their absence is common in real-world, emergent case management and does not invalidate the observed causal association. This limitation highlights an important area for future systematic research into biomarkers for ICIs-related severe cutaneous adverse reactions.

## 4 Conclusion

This case confirms the potential of Sintilimab, a widely used PD-1 inhibitor, to induce life-threatening TEN, even with a favorable antitumor response. It underscores that early recognition, immediate drug cessation, and aggressive supportive care—including immunomodulation and meticulous wound management—are critical for survival. This report highlights the severe risk-benefit dilemma that can arise with ICIs. Further research is needed to identify predictive biomarkers and optimize management protocols for such rare but fatal irAEs.

## Data Availability

The original contributions presented in the study are included in the article/Supplementary Material, further inquiries can be directed to the corresponding author.
