# Supplementary material for: Case Report: Toxic epidermal necrolysis induced by sintilimab in a patient with advanced lung squamous cell carcinoma
Source: Front Pharmacol. 2026 Jan 29;17:1610305. doi: 10.3389/fphar.2026.1610305 (PMC12895052; doi:10.3389/fphar.2026.1610305)
Supplement: Supplementary file 4 [file Table1.docx]

**Supplemental Table 1****. Summary of reported cases of Sintilimab-induced TEN in** **patients with non-small cell lung cancer**

| Feature | Current Case (2024) | Case 1 (Jiang et al., 2023) | Case 2 (Li et al., 2022) | Case 3 (Lye et al., 2023) |
| --- | --- | --- | --- | --- |
| Age (years) / Sex | 60 / female | 68 / male | 59 / male | 72 / male |
| Cancer Type | centrally located lung squamous cell carcinoma | lung adenocarcinoma | centrally located lung squamous cell carcinoma | lung squamous cell carcinoma, poorly differentiated |
| Stage | IV (cT4N3M1, with liver, bone, and cervical lymph node metastasis) | IV (with liver and mediastinal lymph node  Metastasis) | IIIB (T3N0M0) | IV (with hepatic, cerebral, adrenal metastases) |
| Comorbidities | hypertension | none specified | pulmonary tuberculosis (active, on concurrent anti-TB therapy) | none specified |
| Sintilimab Dose | 200 mg | 200 mg | 200 mg | 200 mg |
| Therapy Line & Setting | second-line (post-platinum failure) | first-line (with chemotherapy) | neoadjuvant & adjuvant (with chemotherapy) | first-line (with chemotherapy) |
| Concurrent Therapy | Vinorelbine, palliative RT | Pemetrexed, Cisplatin | Paclitaxel, Cisplatin, Anti-TB drugs | Paclitaxel, Carboplatin |
| Latency to Onset | 3 days after second dose | 7 days after first dose | 10 days after postoperative adjuvant dose | 17 days after first dose |
| Max. BSA Involvement | >85% | not specified (diagnosed as SJS/TEN) | >50% | >30% |
| Mucosal Involvement | ocular, oral, nasal, genital, perianal | oral mucositis | oral mucositis | oral, genital, perianal |
| Diagnostic Method | clinical + multi-disciplinary consult | clinical | clinical | clinical |
| Causality Assessment (Drug) | ALDEN (+4, “Probable”) for Sintilimab | Naranjo (6, “Probable”) for Sintilimab | ALDEN (“Probable”) for Sintilimab | ALDEN (+4, “Probable”) for Sintilimab |
| Key Interventions | high-dose IV MP, IVIG, specialized wound care, antibiotics | oral & IV corticosteroids, antihistamines, topical steroids | IV methylprednisolone, oral prednisone, antibiotics | high-dose IV glucocorticoids, supportive care, wound care |
| Skin Recovery Time | 39 days | 40 days | 72 days | 32 days |
| Antitumor Response | significant tumor reduction post-TEN | 11% tumor reduction post-TEN | partial response (PR) after neoadjuvant therapy | not specified |
| Follow-up & Status | recovered, discharged | not specified | recovered, continued anti-TB therapy | recovered, discharged |
