# Supplementary material for: Case Report: Toxic epidermal necrolysis induced by sintilimab in a patient with advanced lung squamous cell carcinoma
Source: Front Pharmacol. 2026 Jan 29;17:1610305. doi: 10.3389/fphar.2026.1610305 (PMC12895052; doi:10.3389/fphar.2026.1610305)
Supplement: Supplementary file 5 [file Table2.docx]

**Supplemental Table** **2. Radiotherapy plan and outcome summary**

| Parameter | First Course | Second Course |
| --- | --- | --- |
| Time | March 5 to March 21 | March 25 to March 29 |
| Radiation Site | Lumbosacral vertebral bone metastases (L4, L5, S1-S4) | Nasal cavity (nasal septum) tumor and bilateral submandibular lymph nodes |
| Intent | Palliative (for pain relief) | Palliative (for tumor reduction) |
| Technique | Conventional IMRT, CBCT-guided | Conventional IMRT, CBCT-guided |
| Target & Dose | CTV1 (GTV+5mm): 3120 cGy / 13 fx (240 cGy/fx)  PTV1 (CTV+5mm): 2730 cGy / 13 fx (210 cGy/fx) | PGTV1 (GTV1+3-10mm): 1250 cGy / 5 fx (250 cGy/fx) |
| Energy | 6 MV X-ray | 6 MV X-ray |
| Outcome | Significant pain relief. | Treatment discharged due to generalized rash and pruritus. |
