# Supplementary material for: Case Report: Toxic epidermal necrolysis induced by sintilimab in a patient with advanced lung squamous cell carcinoma
Source: Front Pharmacol. 2026 Jan 29;17:1610305. doi: 10.3389/fphar.2026.1610305 (PMC12895052; doi:10.3389/fphar.2026.1610305)
Supplement: Supplementary file 6 [file Table3.docx]

**Supplemental Table** **3. Changes in major laboratory indicators during admission**

| Date | WBC  (10*^9^/L) | CRP  (mg/L) | NEUT  (%) | ALT  (U/L) | IL-6  （pg/ml） |
| --- | --- | --- | --- | --- | --- |
| Reference Range | 4-10 | 0-10 | 40-75 | 5-35 | 0-7 |
| Feb 28 | 9.24 | 38.87 | 72.1 | 17.4 | 21.97 |
| Apr 1 | 2.17 | 178.25 | 79.1 | 19.4 | - |
| Apr 6 | 4.22 | 96.34 | 84.6 | 51.4 | - |
| Apr 14 | 11.20 | >200 | 91.8 | 97.9 | - |
| May 4 | 10.20 | 135.67 | 79.4 | 76 | - |
