# Supplementary material for: Case Report: Toxic epidermal necrolysis induced by sintilimab in a patient with advanced lung squamous cell carcinoma
Source: Front Pharmacol. 2026 Jan 29;17:1610305. doi: 10.3389/fphar.2026.1610305 (PMC12895052; doi:10.3389/fphar.2026.1610305)
Supplement: Supplementary file 7 [file Table4.docx]

**Supplemental Table 4. SCORTEN Assessment for the patient**

| SCORTEN Parameter | Criteria for 1 Point | Patient Data | Point |
| --- | --- | --- | --- |
| Age | ≥ 40 years | 60 years old | 1 |
| Malignancy | Presence of cancer or hematologic malignancy | Stage IV lung squamous cell carcinoma | 1 |
| Heart Rate | ≥ 120 beats per minute (bpm) | 108 bpm | 0 |
| Epidermal Detachment | > 10% of body surface area (BSA) | 11% BSA | 1 |
| Blood Urea Nitrogen (BUN) | > 28 mg/dL (> 10 mmol/L urea) | 18.9 mg/dL | 0 |
| Serum glucose | > 14 mmol/L | 14.52 mmol/L | 1 |
| Bicarbonate | ≤ 20 mmol/L | 29.7 mmol/L | 0 |
| Total SCORTEN Score | | | 4 |
