# Supplementary material for: Case Report: Toxic epidermal necrolysis induced by sintilimab in a patient with advanced lung squamous cell carcinoma
Source: Front Pharmacol. 2026 Jan 29;17:1610305. doi: 10.3389/fphar.2026.1610305 (PMC12895052; doi:10.3389/fphar.2026.1610305)
Supplement: Supplementary file 8 [file Table5.docx]

**Supplemental Table 5.** **Comparative ALDEN-based causality assessment of suspected agents**

| ALDEN Criterion & Values | Sintilimab (anti-PD-1) | Vinorelbine (Oral) | Radiotherapy | Fluconazole |
| --- | --- | --- | --- | --- |
| 1. Chronology of Drug to Onset | Started Mar 25; rash onset Mar 28 (3-day lag). Value: +1 (Likely) | Started Mar 25; rash onset Mar 28 (3-day lag). Value: +1 (Likely) | Lumbosacral RT ended Mar 21 (7-day lag). Nasal RT concurrent. TEN post-RT is atypical. Value: 0 (Neutral/Unlikely) | Started Mar 31, after rash onset (index day). Value: –3 (Excluded) |
| 2. Drug Presence in Body | Present (long half-life ~15-20 days). Value: 0 (Definite) | Present. Value: 0 (Definite) | Not applicable in classical typical pharmacokinetics terms. Value: 0 (Neutral) | Not applicable. |
| 3. Prechallenge/Rechallenge | Not performed. Value: 0 (Not done) | Not performed. Value: 0 (Not done) | Not performed. Value: 0 (Not done) | Not applicable. |
| 4. Dechallenge | **Drug stopped.** **Value: 0 (Neutral)** | **Drug stopped.** **Value: 0 (Neutral)** | **Treatment stopped.** **Value: 0 (Neutral)** | Not applicable. |
| 5. Drug Notoriety | High-risk drug class for SCARs/SJS/TEN per pharmacovigilance. Value: +3 (Strongly associated) | Extremely rare association with TEN for oral formulation. Value: 0 (Unknown) | Atypical cause for systemic TEN. Value: 0 (Unknown) | Not applicable. |
| 6. Other Cause | Intermediate Value of sintilimab >3. No other drug Values higher. Value: 0 | At least one other drug (Sintilimab) has an intermediate Value >3. Value: –1 | At least one other drug (Sintilimab) has an intermediate Value >3. Value: –1 | Excluded. |
| Intermediate Value (Sum of Criteria 1-5) | **(+1) + 0 + 0 + 0 + (+3) = +4** | **(+1) + 0 + 0 + 0 + 0 = +1** | **0 + 0 + 0 + 0 + 0 = 0** | Excluded |
| Final ALDEN Value (Intermediate + Criterion 6) | **+4** | **0** | **–1** | Excluded |
| Interpreted Causality | **Probable** | **Unlikely** | **Very Unlikely** | Excluded |

*ALDEN causality assessment was performed according to the standard algorithm (Sassolas et al., 2010).*
